# Degradation of 4-chloro-3-nitrophenol via a novel intermediate, 4-chlororesorcinol by *Pseudomonas* sp. JHN

**DOI:** 10.1038/srep04475

**Published:** 2014-03-26

**Authors:** Pankaj Kumar Arora, Alok Srivastava, Vijay Pal Singh

**Affiliations:** 1 Department of Plant Science, Faculty of Applied Sciences, MJP Rohilkhand University, Bareilly, India

## Abstract

A 4-chloro-3-nitrophenol (4C3NP)-mineralizing bacterium, *Pseudomonas* sp. JHN was isolated from a waste water sample collected from a chemically-contaminated area, India by an enrichment method. *Pseudomonas* sp. JHN utilized 4C3NP as a sole carbon and energy source and degraded it with the release of stoichiometric amounts of chloride and nitrite ions. Gas chromatography and gas chromatography-mass spectrometry detected 4-chlororesorcinol as a major metabolite of the 4C3NP degradation pathway. Inhibition studies using 2,2′-dipyridyl showed that 4-chlororesorcinol is a terminal aromatic compound in the degradation pathway of 4C3NP. The activity for 4C3NP-monooxygenase was detected in the crude extracts of the 4C3NP-induced JHN cells that confirmed the formation of 4-chlororesorcinol from 4C3NP. The capillary assay showed that *Pseudomonas* sp. JHN exhibited chemotaxis toward 4C3NP. The bioremediation capability of *Pseudomonas* sp. JHN was monitored to carry out the microcosm experiments using sterile and non-sterile soils spiked with 4C3NP. Strain JHN degraded 4C3NP in sterile and non-sterile soil with same degradation rates. This is the first report of (i) bacterial degradation and bioremediation of 4C3NP, (ii) formation of 4-chlororesorcinol in the degradation pathway of 4C3NP, (iii) bacterial chemotaxis toward 4C3NP.

Chloronitrophenols (CNPs) are a class of chlorinated nitroaromatic compounds, which are used in the production of fungicides, dyes and substrates for various enzyme activities^1^,^2^. Examples are 2-chloro-4-nitrophenol (2C4NP), 4-chloro-2-nitrophenol (4C2NP), 4-chloro-3-nitrophenol (4C3NP), 2-chloro-3-nitrophenol (2C3NP) and 2-chloro-5-nitrophenol (2C5NP)^1^. CNPs have been introduced into the environment because of anthropogenic activities^2^,^3^,^4^.

Microbial degradation of CNPs has been investigated in a few bacteria and two routes of degradation of CNPs have been identified: oxidative and reductive^1^,^2^,^3^,^4^,^5^,^6^,^7^,^8^,^9^,^10^,^11^,^12^. In the oxidative pathway, CNPs were degraded with oxidative removal of nitrite ions and followed by removal of chloride ions and/or ring-cleavage^5^,^6^,^8^,^10^,^11^. In the reductive pathway, degradation of CNPs were initiated either reduction of nitro group into ammonia or reductive removal of chloride ions^2^,^3^,^4^.

*Arthrobacter nitrophenolicus*^5^,^11^, *Burkholderia* sp. SJ98^8^, *Burkholderia* sp. RKJ 800^12^ and *Rhodococcus imtechensis* RKJ300^6^ utilized 2C4NP as the sole carbon and energy source and degraded it via different pathways. *Arthrobacter nitrophenolicus* SJCon degraded 2C4NP via chlorohydroquinone (CHQ) and maleylacetate^5^ whereas strains RKJ 800 and RKJ300 degraded 2C4NP via the formation of CHQ and hydroquinone^6^,^12^. Another 2C4NP-degrading bacterium, *Burkholderia* sp. SJ98 degraded 2C4NP via 4-nitrophenol, 4-nitrocatechol and 1,2,4-benzenetriol^8^.

The complete degradation of 4C2NP was also studied in *Exiguobacterium* sp. PMA, which utilized it as a sole carbon and energy source and degraded it with the formation of 4-chloro-2-aminophenol (4C2AP) and 2-aminophenol^2^. Another bacteria, *Bacillus* sp. RKJ 700^3^ and *Bacillus subtilis* RKJ 600^4^ completely biotransformed 4C2NP into 5-chloro-2-methyl benzoxazole via the formation of 4C2AP and 4-chloro-2-acetaminophenol. A co-culture of two bacteria degraded 4C2NP under coupled oxidative and reductive conditions^9^. A genetically engineered bacterium *Pseudomonas* sp. N31 also utilized 4C2NP as a sole source of carbon and energy and degraded it via chlorocatechol^11^.

In this study, we have selected 4C3NP as a model compound for the studies of degradation of CNPs. Although, several CNPs-degrading bacteria have been isolated; however, there is no report of bacterial degradation of 4C3NP. The present communication describes: (i) the isolation of an efficient 4C3NP mineralizing bacterial strain; (ii) the identification of metabolite of degradation of 4C3NP by isolate; (iii) the bioremediation of 4C3NP in the soil using isolate; and (iv) the chemotaxis toward 4C3NP.

## Results

### Identification of a 4C3NP-degrading bacterium

A 4C3NP-mineralizing bacterial strain JHN was isolated from a waste water sample collected from a chemically-contaminated area, India and identified as *Pseudomonas* sp. on the basis of the 16S rRNA gene sequence analysis. The 16S rRNA gene sequence of *Pseudomonas* sp. JHN was submitted at the GenBank under the accession number KC733809.

### Growth and degradation studies

**Figure 1:**
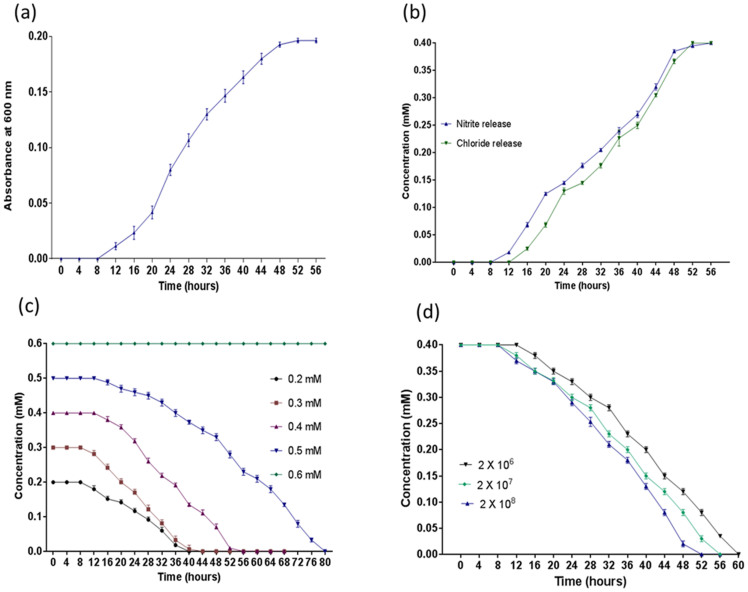
Growth and Degradation Studies. (a) Growth of *Pseudomonas* sp. JHN on 4C3NP. (b) Analysis of chloride and nitrite releases from the 4C3NP degradation by *Pseudomonas* sp. JHN. (c) Effect of various concentrations of 4C3NP on the 4C3NP degradation by *Pseudomonas* sp. JHN. (d) Effects on different inoculum sizes on degradation of 4C3NP by *Pseudomonas* sp. JHN.

For growth studies, strain JHN was grown on minimal media containing 0.4 mM 4C3NP as the sole carbon and energy source. The growth of strain JHN was monitored spectrophotometric analysis with increase the optical density at 600 nm and the maximum absorbance of growth was recorded as 0.194 (Fig. 1a). Strain JHN degraded 4C3NP completely within 52 h and the stoichiometric amounts of chloride and nitrite ions were detected during the degradation of 4C3NP (Fig. 1b). No ammonia release was observed during the degrading studies. These data suggest that degradation of 4C3NP occurred via oxidative removal of nitrite ions.

*Pseudomonas* sp. JHN degraded 4C3NP when the range of the concentration was from 0.1 mM to 0.5 mM (Fig. 1c). Strain JHN was not able to degrade 0.6 mM 4C3NP. The optimum concentration for degradation of 4C3NP by strain JHN was determined as 0.4 mM on the basis of rapid degradation rate and optimum growth at this concentration.

The effect of various inoculum sizes on the 4C3NP degradation was also determined and it was observed that the rate of the 4C3NP degradation was faster in a culture inoculated with highest densities as compared to both of the cultures having lower cell densities (Fig. 1d).

### Identification of metabolite

**Figure 2:**
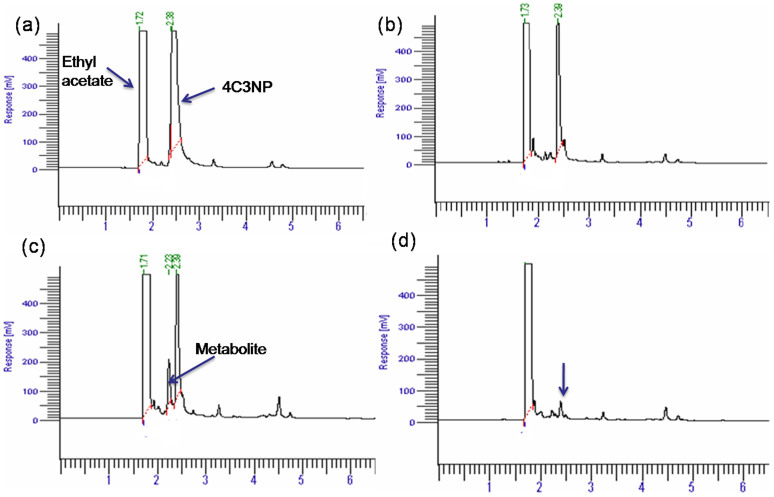
Gas Chromatography elution profile of samples of degradation of 4C3NP by *Pseudomonas* sp. JHN.

**Figure 3:**
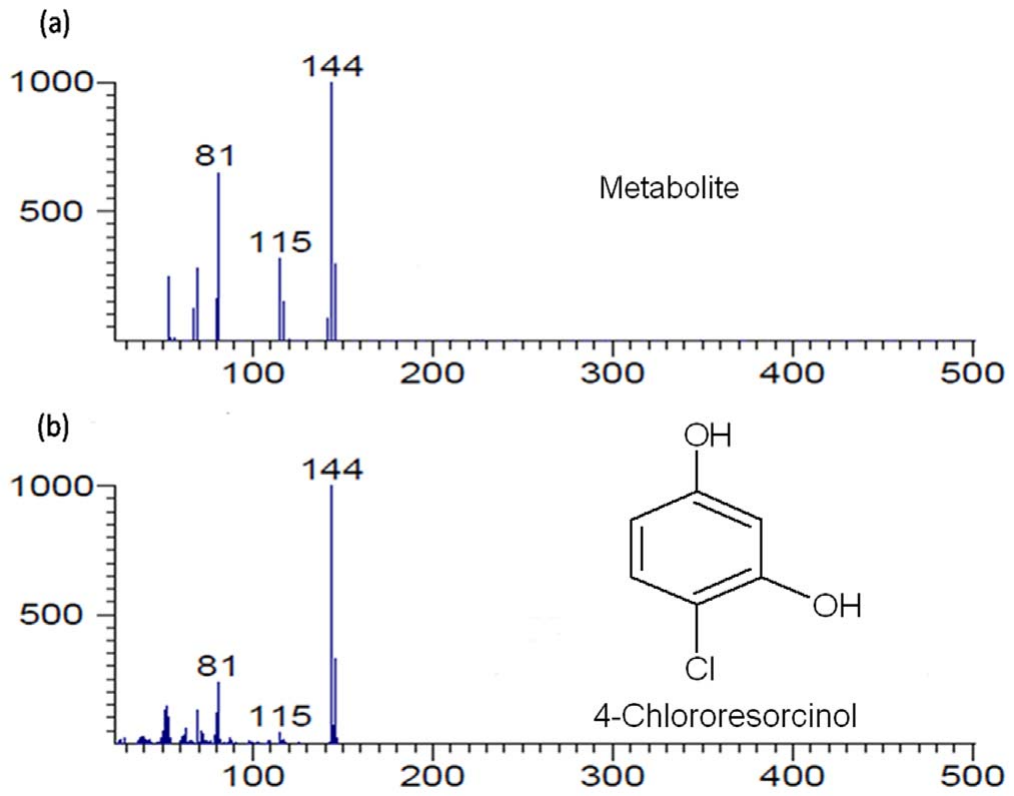
Mass spectrum of metabolite I (a) and authentic standard (b).

The gas chromatography (GC) and gas chromatography-mass spectrometry (GC-MS) studies were carried out to monitor the degradation of 4C3NP by strain JHN. GC analysis confirmed complete depletion of 4C3NP by strain JHN within 50 h (Fig. 2). In 12 h sample, a peak of 4C3NP was detected along with the peak of the ethylacetate in which the sample was dissolved (Fig. 2a). The retention time of the peaks of 4C3NP and ethylacetate were 2.39 and 1.72 min. respectively. In the sample of 24 h, the depletion of 4C3NP was observed (Fig. 2b). In the sample of 36 h, a peak of the metabolite (retention time 2.23 min) was observed along with the peaks of 4C3NP and ethylacetate (Fig. 2c). In the sample of 48 h, the peak of metabolite disappeared and the 4C3NP was detected in minor quantities (Fig. 2d) which completely disappeared in the sample of 50 h (data not shown). To identify the metabolite, the GC-MS was carried out. The mass spectrum of metabolite was observed at 144 m/z that is identical to authentic standard 4-chlororesorcinol (Fig. 3). On the basis of these results, metabolite was identified as 4-chlororesorcinol.

### Inhibition studies

**Figure 4:**
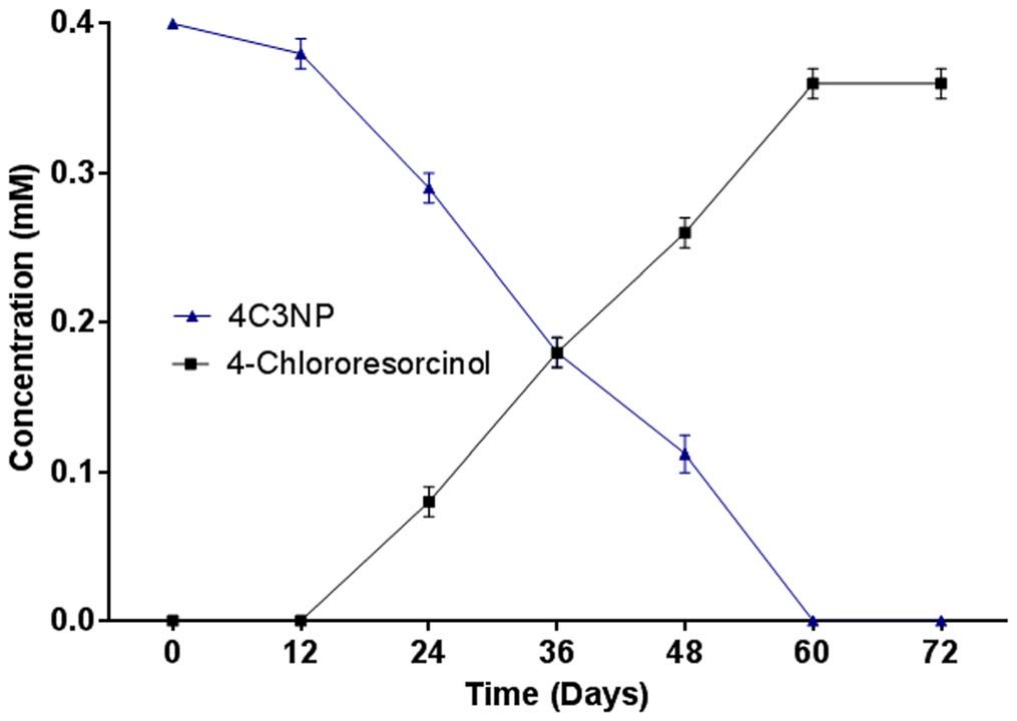
Accumulation of 4-chlororesorcinol during the inhibition studies.

The results of inhibition studies showed the accumulation of the 4-chlororesorcinol into the culture medium due to the blockage of the ring-cleavage activity of the enzyme chlororesorcinol dioxygenase by 2, 2′-dipyridyl (Fig. 4). On the basis of this experiment, we concluded that 4-chlororesorcinol is a terminal aromatic compound in the degradation pathway of 4-chlororesorcinol.

### Enzyme assay for 4C3NP-3-monooxygenase

The 4C3NP-3-monooxygenase catalyzed the conversion of 4C3NP into 4-chlororesorcinol with the release of nitrite ions. GC-MS detected 4-chlororesorcinol in the extracted sample collected after the incubation of the reaction mix at room temperature for 15 min. The stoichiometric amounts of nitrite ions were detected in non-extracted sample. Neither 4-chlororesorcinol nor nitrite ions were detected in the control.

### Enzyme assay for 4-chlororesorcinol dioxygenase

The 4-chlororesorcinol dioxygenase catalyzes the conversion of 4-chlororesorcinol into chlorohydroxymuconic semialdehyde. The spectrophotometric assays showed decrease of the wavelength at 280 nm crossponding to 4-chlororesorcinol and increase of the wavelength at 375 nm crossponding to chlorohydroxymuconic semialdehyde. However, we cannot detect chlorohydroxymuconic semildehyde by GC-MS analysis due to the instability of this compound. On the basis of the chlororesorcinol dioxygenase assay, we assume that 4-chlororesorcinol may cleave into an isomer of chlorohydroxymuconic semialdehyde by chlororesorcinol dioxygenase.

### Microcosm studies

**Figure 5:**
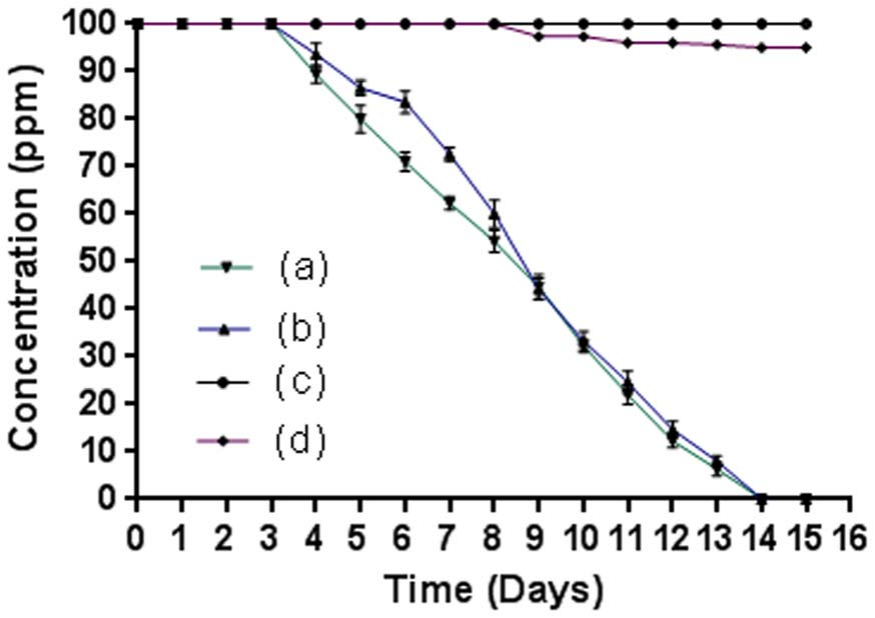
Microcosm studies. (a) Degradation of 4C3NP in sterile soil by *Pseudomonas* sp. JHN. (b) Degradation of 4C3NP in non-sterile soil by by *Pseudomonas* sp. JHN. (c) Degradation of 4C3NP in control microcosm with sterile soil, (d) Degradation of 4C3NP in control microcosm with non-sterile soil.

*Pseudomonas* sp. JHN degraded 4C3NP in both sterile and non-sterile soil microcosms under optimized conditions. The optimized conditions were as follows: inoculum size 2 × 10^7^ CFU g^−1^ soil, pH 7.5, temperature 30°C, and substrate concentration 100 ppm of 4C3NP. Strain JHN completely degraded 4C3NP in the test microcosm with sterile soil within 14 days (Fig. 5a). There was no degradation at initial three days after incubation. At 4^th^ days, 8% degradation was observed and degradation was 35% by 7^th^ days. At 9 days, almost 60% degradation of 4C3NP was completed. The degradation was 85% by 12 days. Almost complete degradation of 4C3NP was observed at 14 days. In another test microcosm with non-sterile soil, the complete 4C3NP depletion occurred within 14 days (Fig. 5b). In controls with sterile and non-sterile soils, no degradation was observed within 15 days (Fig. 5c and 5d).

### Chemotaxis towards 4C3NP

**Figure 6:**
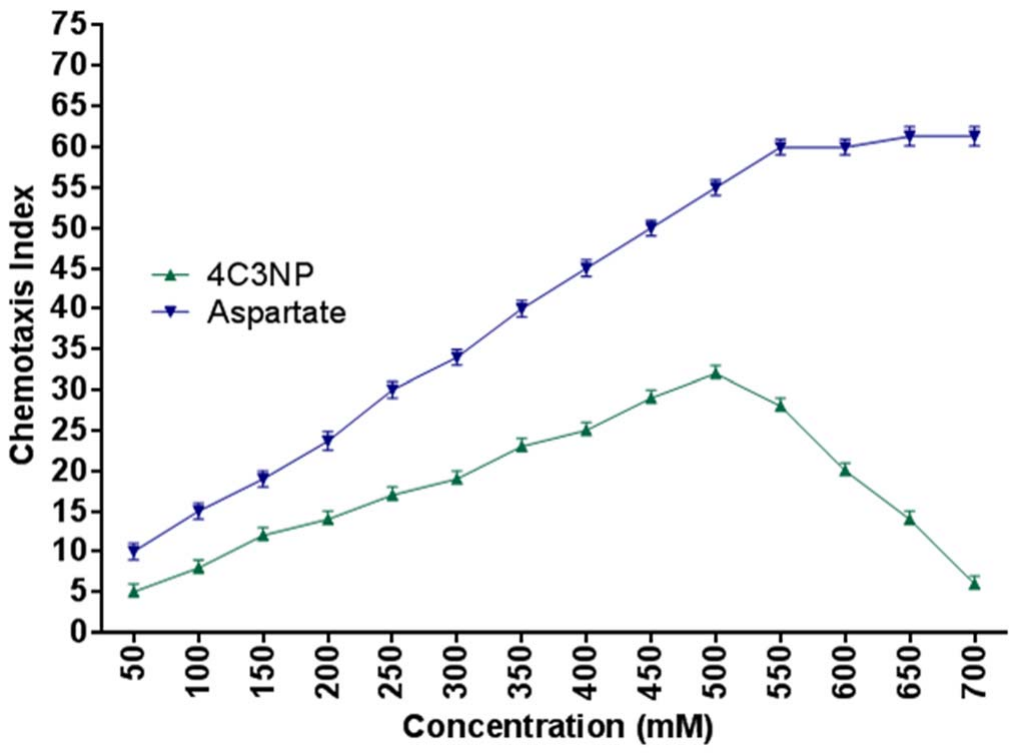
Chemotaxis of *Pseudomonas* sp. JHN toward 4C3NP using capillary assay.

Chemotaxis of strain JHN toward 4C3NP was determined by the capillary assay. In capillary assay, it was observed that the cells of strain JHN were chemotactic towards 4C3NP at an optimum concentration of 500 μM with a chemotaxis index (C.I.) of 32. Figure 6 showed that the C.I value gradually increased with increased the concentration of 4C3NP until optimal concentration (500 μM). After optimum concentration, C.I values are decreased for 4C3NP. Aspartate was a positive control and there was no decrease of C.1 values.

## Discussion

An efficient 4C3NP-mineralizing bacterium, *Pseudomonas* sp. JHN degraded 4C3NP up to a concentration of 0.5 mM. The stoichiometric amounts of chloride and nitrite ions were released from 4C3NP by strain JHN. 4-Chlororesorcinol was identified as a metabolite of the 4C3NP degradation pathway in *Pseudomonas* sp. JHN. This is the first report of the formation of 4-chlororesorcinol in the degradation pathway of 4C3NP. Bian et al.^15^ reported formation of 4-chlororesorcinol as an intermediate product of degradation of 4-chlorophenol in water by pulsed high voltage discharge. Boyd et al.^16^ studied the reductive dechlorination of 4-chlororesorcinol to resorcinol by anaerobic bacteria. The enzymatic transformation of 4-chlororesorcinol to hydroxyquinol has also been reported^17^. In this study, neither resorcinol nor hydroxyquinol was detected as the intermediate product of degradation of 4C3NP, suggesting that these intermediates should not involve in the degradation pathway of 4C3NP.

**Figure 7:**
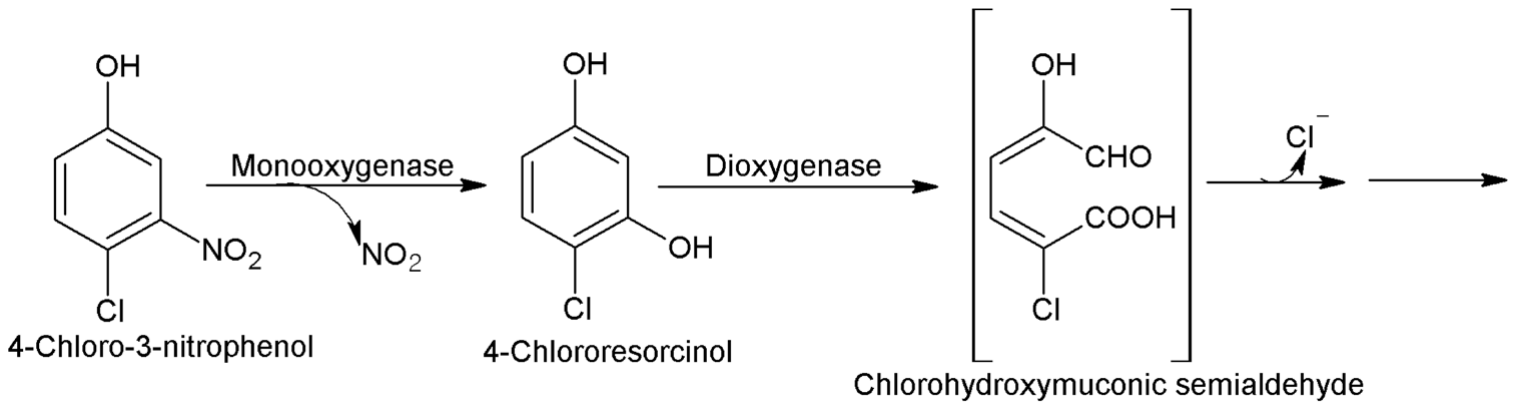
Degradation of 4C3NP by bacteria. Proposed pathway of degradation of 4C3NP for *Pseudomonas* sp. JHN.

The initial mechanism of degradation of 4C3NP by strain JHN was similar to the degradation of 4C2NP and 2C4NP by other bacteria. A genetically engineered bacterium, *Pseudomonas* sp. N-31 degraded 4C2NP by the oxidative removal of nitro group and formation of 4-chlorocatechol, which was further degraded with release of chloride ions^10^. In this study, *Pseudomonas* sp. JHN degraded 4C3NP by the similar mechanism with the oxidative removal of nitrite group and formation of 4-chlororesorcinol which was further degraded with release of chloride ion. Another bacterium, *Arthrobacter nitrophenolicus* SJCon degraded 2C4NP with the oxidative removal of nitro group, followed by the release of chloride ions^5^. It was observed that a specific monooxygenase should be involved in the initial step of the degradation of 4C3NP and other CNPs. We have detected the activity of 4C3NP-monooxygenase in the crude extracts of the 4C3NP-induced cells of strain JHN. Similarly, Arora and Jain^12^ detected the 4C2NP- monooxygenase activity in the degradation pathway of 2C4NP in *Burkholderia* sp. RKJ 800. On the basis of the above discussion, we conclude that CNPs-degrading bacteria have a common mechanism for degradation of various CNPs. We have also observed the enzymatic activity for chlororesorcinol dioxygenase that suggested the cleavage of 4-chlororesorcinol to an isomer of chlorohydroxymuconic semialdehyde. On the basis of the identified metabolites as well as enzyme assays, we have proposed a new pathway for degradation of 4C3NP for *Pseudomonas* sp. JHN. The degradation was initiated with release of nitrite ion and formation of 4-chlororesorcinol by an enzyme 4C3NP-monooxygenase. In the next step, 4-chlororesorcinol dioxygenase cleaved 4-chlororesorcinol into an isomer of chlorohydroxymuconic semialdehyde that may be degraded with release of chloride ion [Fig. 7].

The bioremediation capacity of strain JHN to degrade 4C3NP in soil was monitored to carry out microcosm studies with 4C3NP-spiked sterile and non-sterile soil. Strain JHN completely degraded 4C3NP in sterile and non-sterile soil microcosms almost with equal rate. These data suggest that there is no effect on the 4C3NP degradation rate by indigenous bacteria. It may also possible that the growth of indigenous bacteria may be inhibited due to toxic effects of 4C3NP. In the control (sterile and non- sterile microcosm without inoculation), there was no degradation of 4C3NP suggesting that strain JHN was involved in the degradation of 4C3NP and there is no role of internal or biotic factors on the degradation. This is the first report of the degradation of 4C3NP in soil. Previously, several bacteria have been shown degradation of other CNPs (2C4NP and 4C2NP) in soil^2^,^6^,^12^. *Rhodococcus imtechensis* RKJ300^6^ and *Burkholderia* sp. RKJ 800^12^ degraded 2C4NP in the soil microcosm. However, the rate of the degradation of 2C4NP was faster in non-sterile soil as compared to sterile soil. Similar results were obtained in the case of *Exiguobacterium* sp. PMA, which degraded 4C2NP rapidly in non-sterile soil as compared to sterile soil^2^. In this study, strain JHN degraded 4C3NP in non-sterile soil as well as sterile soil with same rate.

*Pseudomonas* sp. JHN degraded 4C3NP and exhibited a positive chemotactic response toward it. This property may increase the bioremediation potential of strain JHN by increasing the 4C3NP bioavailability to this bacterium in soil. Previously, few bacteria exhibited chemotaxis toward CNPs. For example, *Burkholderia* sp. strain SJ98 exhibited chemotactic response toward few CNPs including 2C4NP and 2C3NP^18^,^19^.

## Methods

### Isolation and identification of a 4C3NP-degrading bacterium

A 4C3NP-degrading bacterium, *Pseudomonas* sp. JHN was isolated from the waste water collected from a chemically-contaminated area, India by an enrichment method using 4C3NP as a substrate. For enrichment, 2 ml of the waste-water sample was added to 500 ml Erlenmeyer flask containing 100 ml minimal media and 0.2 mM 4C3NP as a sole carbon and energy source. The composition of minimal medium was exactly same as described previously^2^,^3^,^4^. The enrichment flask was incubated at 30°C under shaking conditions at 200 rpm. Upon decolourization, cultures were serially diluted in minimal media agar plates containing 0.2 mM 4C3NP. About ten different morphotypes were grown on the minimal agar plates containing 0.2 mM 4C3NP. Only one colony showed decolourization around it suggesting that this bacterium has ability to utilize 4C3NP. This colony was streaked further on nutrient agar plate to check the purity and pure culture designated as strain JHN was preserved in 10% glycerol vial at −80°C.

To identify the strain JHN, the 16S rRNA gene sequence of strain JHN was amplified using the universal primers, 27F (5′-AGAGTTTGATCCTGGCTCAG-3′) and 1492R (5′-TACGGYTACCTTGTTACGACTT-3′) and sequenced using Big Dye terminator cycle sequencing ready reaction kit (Applied Biosystems) by an automated DNA sequencer (ABI 3130 XL Genetic Analyzer; Applied Biosystems)^11^,^13^,^14^. The 16S rRNA gene sequence of strain JHN was determined by using the programme BLAST.

### Growth and degradation studies

The growth and degradation studies were carried out using minimal media containing 0.4 mM 4C3NP as a sole carbon and energy source. The ability of strain JHM to utilize 4C3NP as a sole carbon and energy source was determined by monitoring the optical density of the bacterial culture at 600 nm using a spectrophotometer. The depletion of 4C3NP was checked by monitoring the absorbance at 420 nm. The chloride, ammonia and nitrite ions were analyzed by previously described methods^5^,^12^.

The effect of various substrate concentrations on the 4C3NP degradation were determined using minimal media containing desired concentration of 4C3NP (0.2 mM, 0.3 mM, 0.4 mM, and 0.5 mM). Samples were collected at regular intervals and degradation was monitored by spectrometric method as described above.

The effect of different inoculums sizes on the 4C3NP degradation was determined on minimal media containing 0.4 mM 4C3NP using bacterial cultures with different densities (2.0 × 10^6^, 2.0 × 10^7^, and 2 × 10^8^ CFU/ml). The 4C3NP degradation was monitored as described above.

### Identification of metabolites

Samples were collected at regular intervals (12h, 24h, 36h, 48h, 50h) from the bacterial broth culture containing 0.4 mM 4C3NP and centrifuged at 10000 X. The supernatants were extracted with ethyl acetate and the extracted samples were analyzed by analytical techniques.

### Gas-Chromatography (GC)

The GC analysis was carried out using AutoSystem-XL Gas chromatograph (Perkin-Elmer Inc. Waltham, MA, USA) equipped with flame-ionization detector. Temperatures for injector, oven, and detector were kept constant at 280°C, 200°C, and 250°C, respectively. The peak of parent compound and metabolite were identified on the basis of the comparison with the retention time of standard compounds.

### Gas chromatography and mass spectrometry (GC-MS)

To confirm the identification of the metabolite, the samples were analyzed with GC-MS. Agilent Gas Chromatography system model 7890A connected to model Pegasus*®* High Throughput Time-of-Flight Mass Spectrometer (HT-TOFMS) from Leco was used with a HP-5 (30 m × 0.320 mm × 0.25 μm) column. Separation was performed under the following temperature program on the low-polarity columns (HP-5 ms) with helium as a carrier gas at 1.5 mL/min; 50°C held for 1 min and the temperature was increased at 25°/min to 280°C and held for 5 min. The samples (1 μL) were injected in splitless mode. The temperatures of the transfer line and ion source (electron ionisation mode, EI, 70 eV) were 225°C and 250°C, respectively.

### Inhibition studies

Strain JHM was grown in the minimal medium containing 10 mM sodium succinate, 0.4 mM 4C3NP and 1.5 mM 2,2′-dipyridyl. Samples were collected at regular intervals and analyzed by the gas chromatography as described above.

### 4-Chloronitrophenol-3-monooxygenase activity

4-Chloronitrophenol-3-monooxygenase (CNP-3-monooxygenase) activity was determined by measuring the formation of 4-chlororesorcinol and nitrite ions from 4C3NP. The reaction mixture (1 ml volume) was comprised of 50 mM Tris–Cl (pH 8.0), 0.2 mM NADH, 0.08 mM FAD, crude extracts, and 400 μM 4C3NP. Crude extracts used in this study was prepared using 4C3NP as described previously^2^. In the control experiment, crude extracts were heated at 100°C before adding to the reaction mixture. The samples were collected after 15 min of incubation at room temperature and centrifuged. The supernatants were subjected for detection of the nitrite ions by the previously described method^5^,^12^. The supernatants were extracted with ethyl acetate and analyzed by GC-MS for identification of 4-chlororesorcinol.

### Chlororesorcinol dioxygenase activity

The activity for chlororesorcinol dioxygenase was determined spectrophotometrically by monitoring the depletion of 4-chlororesorcinol at 280 nm and the formation of chlorohydroxymuconic semialdehyde at 375 nm. Enzyme assay was carried out to incubate the 5 ml reaction mixture at room temperature. The reaction mixture was comprised of 50 mM Tris-Cl, 0.2 mM 4-chlororesorcinal, 0.2 mM ferrous sulfate and crude extracts. Samples were taken at 0, 3, 6, 8, and 10 min and analyzed by a spectrophotometer.

### Microcosm studies

Microcosms were prepared with glass beaker (of 500 ml volume each) by distributing 100 grams of soil spiked with 70 ppm 4C3NP as following: (a) test microcosm with sterile soil, (b) test microcosms with non-sterile soil, (c) control microcosm with sterile soil, and (b) control microcosm with non-sterile soil. Both of test microcosms were mixed with pre-grown 4C3NP-induced JHN cells at ~2 × 10^7^ cells colony-forming units (CFUs) g^−1^ soil. Both of the control microcosms were non-bioaugmented during the whole microcosm studies^2^. All the soil microcosms covered with perforated aluminium foil were incubated at 30°C for 15 days. The water contents in all the microcosms were monitored during the incubation period and maintained by sprinkling with distilled water at regular intervals^2^. The optimum conditions (optimum inoculum size, optimum pH, adequate temperature and suitable substrate concentration) for the effective degradation of 4C3NP in the soil microcosm were determined prior to the study as described previously^2^.

### Chemotaxis towards 4C3NP

The chemotactic response of *Pseudomonas* sp. JHN towards 4C3NP was investigated with capillary assays as described by Pandey et al.^18^. The optimum concentration of 4C3NP for capillary assay was determined by performing assays at various 4C3NP concentrations (from 50–700 μM in 50 μM increments)^18^. First, glass capillaries (10 μl) were filled with the chemotaxis solution containing chemotactic compound (in desired concentration), 100 mM potassium phosphate (pH 7.0) and 20 μM EDTA). The control capillaries were without any chemotactic compound. Capillaries were then inserted into a glass slide containing a suspension (10^13^ cells/ml) of cells of strain JHN, and incubated at room temperature for 45 min. The solution filled in capillaries were then serially diluted and plated onto nutrient agar. Colony forming units (CFUs) were counted after 24 h incubation at room temperature. Chemotaxis index (ratio of the number of CFUs produced from the capillary containing chemotactic compound to CFUs produced from a control capillary was used to quantify the chemotactic response^18^. Aspartate was used as the positive control.

## Author Contributions

P.K.A. and V.P.S. conceived and designed the experiments. P.K.A. and A.S. performed the experiments, analyzed the data, contributed reagents and materials. P.K.A. and V.P.S. wrote the paper. All authors reviewed the manuscript.
